# Characterisation and development of histopathological lesions in a guinea pig model of *Mycobacterium tuberculosis* infection

**DOI:** 10.3389/fvets.2023.1264200

**Published:** 2023-09-22

**Authors:** Fernanda Larenas-Muñoz, Inés Ruedas-Torres, Laura Hunter, Alison Bird, Irene Agulló-Ros, Rebecca Winsbury, Simon Clark, Emma Rayner, Francisco J. Salguero

**Affiliations:** ^1^Department of Anatomy and Comparative Pathology and Toxicology, Pathology and Immunology Group (UCO-PIG), UIC Zoonosis y Enfermedades Emergentes ENZOEM, University of Córdoba, International Excellence Agrifood Campus ‘CeiA3’, Córdoba, Spain; ^2^Pathology Department, UK Health Security Agency (UKHSA), Porton Down, Salisbury, United Kingdom; ^3^School of Biosciences and Medicine, University of Surrey, Guildford, United Kingdom

**Keywords:** tuberculosis, guinea pig, granuloma, pathology, animal model, immunohistochemistry, cell marker, *Mycobacterium tuberculosis*

## Abstract

Tuberculosis (TB) remains a very significant infectious disease worldwide. New vaccines and therapies are needed, even more crucially with the increase of multi-drug resistant *Mycobacterium tuberculosis* strains. Preclinical animal models are very valuable for the development of these new disease control strategies. Guinea pigs are one of the best models of TB, sharing many features with the pathology observed in human TB. Here we describe the development of TB lesions in a guinea pig model of infection. We characterise the granulomatous lesions in four developmental stages (I–IV), using histopathological analysis and immunohistochemical (IHC) techniques to study macrophages, T cells, B cells and granulocytes. The granulomas in the guinea pigs start as aggregations of macrophages and few heterophils, evolving to larger lesions showing central caseous necrosis with mineralisation and abundant acid-fast bacilli, surrounded by a rim of macrophages and lymphocytes in the outer layers of the granuloma. Multinucleated giant cells are very rare and fibrotic capsules are not formed in this animal model.

## 1. Introduction

Tuberculosis (TB) is a chronic disease which remains a significant global health threat, particularly to immunosuppressed individuals (1, 2). TB accounts for over 1.6 million human deaths worldwide (3), 191.000 of which were due to resistance to antibiotic treatment in 2021 (4). Because of this, animal models have been used historically to gain a better understanding of the disease and evaluate new therapies and vaccines (5).

The tuberculous granuloma is the hallmark lesion induced by *Mycobacterium tuberculosis* complex (MTBC) bacteria (6). These granulomas exhibit similar features in human disease, as well as in both experimental and natural animal infections, with differences dependent on the animal species and type of MTBC bacteria involved. Granulomas are mainly composed of an accumulation of epithelioid macrophages, lymphocytes, some plasma cells and granulocytes which, depending on the chronicity, may include a necrotic centre with or without dystrophic mineralisation, and surrounded by a connective tissue capsule (7–16).

Guinea pigs have been widely used as an animal model for the study of various infectious diseases (17, 18) and have been found to be more susceptible to tuberculosis compared to other animal models (19, 20). Laboratory mice, for example, develop granulomatous lesions that differ significantly from those observed in humans, including the cellular composition as well as the progression to necrosis and lack of mineralisation (7, 19, 21). Non-human primates are very good models of human TB, showing many similarities with the granulomatous lesions observed in humans (15, 22). However, this animal model is costly and, due to its similarity to people and the ethical considerations alongside this, its use is strictly regulated (19, 23). Tuberculous granulomas in rabbits and guinea pigs share many similarities to those observed in humans (7, 20, 23); however, the number of reagents available to evaluate immune responses and cellular composition of granulomas in these species is scarce (19). Immunohistochemistry (IHC) provides additional information to conventional histopathological studies by using specific antibodies against MTBC antigens or cell markers, helping to understand the mechanisms of immune response, granuloma formation and the interaction of the bacteria with the host (8, 24).

It is believed that guinea pigs are thought to be the most susceptible animal model of TB (7) and previous reports have briefly described the development and characterisation of tuberculous granulomas in this species (11, 12, 25–28). Whilst much research has been focused on this model, currently, there is no robust scoring system for assessing the microscopic features of pulmonary granulomas to aid the evaluation of disease severity and progression in studies that assess new vaccines and therapeutics. Furthermore, there is scope to expand the understanding of cellular composition for each granuloma stage using various immunochemical markers, as well as investigating the presence and frequency of mycobacteria. In this study, we have used archived and new material from *Mycobacterium tuberculosis* (Mtb) infected guinea pigs, to characterise and categorise the development of pulmonary granulomas post-infection. Moreover, we have employed a panel of commercially available antibodies to study the different cell populations within the granulomas.

## 2. Materials and methods

### 2.1. Experimental animals

Wherever possible, analyses of existing, formalin-fixed paraffin-embedded (FFPE) lung tissues from an archive of historical experiments (equating to 44 animals), were used in this study to reduce the number of additional animals needed for analyses. Twelve additional adult, female Dunkin Hartley guinea pigs (*Cavia porcellus*), free from pathogen-specific infection, with a body weight of 300–400 g, were obtained from a UK Home Office accredited facility (Envigo, United Kingdom). Animals were randomly assigned to groups and identified using subcutaneously implanted microchips (Plexx, Netherlands) to enable blinding of the analyses. Animals were housed at ACDP (Advisory Committee on Dangerous Pathogens) level 3, post-infection in groups of up to eight, with access to food and water *ad libitum*. The housing environment was maintained within a temperature range of 15–21°C and a relative humidity range of 45 to 65%. Group sizes were determined by statistical power calculations (Minitab, version 16) performed using previous data (SD, approximately 0.5) to reliably detect a difference of 1.0 log_10_ in the median number of colony-forming units (cfu) per millilitre. All animal procedures were approved by the United Kingdom Health Security Agency, Porton Down Establishment Animal Welfare and Ethical Review Body and authorised under an appropriate UK Home Office project license. Animals’ clinical and behavioural status were monitored daily.

### 2.2. Inoculum

The Mtb H37Rv strain was used for the challenge. National Collection of Type Cultures (NCTC) 7,416 challenge stock was generated from a chemostat grown to steady state under controlled conditions at 37°C ± 0.1, pH 7.0 ± 0.1 and a dissolved oxygen tension of 10% ± 0.1, in a defined medium, the details of which have been previously described (29). Aliquots were stored at −80°C. Titre of the stock suspension was determined from thawed aliquots by enumeration of Cfus cultured onto Middlebrook 7H11 OADC selective agar.

### 2.3. Infection

Challenge for each group of animals was by the aerosol route with Mtb strain H37Rv. Animals were challenged using a contained Henderson apparatus in conjunction with an AeroMP control unit as previously described (30, 31). Aerosol particles generated were delivered to the animals via both nares using a 3-jet Collison nebuliser, for an exposure time of 5 min. The challenge suspension in the nebuliser was adjusted by dilution in sterile water to a concentration of between 5 × 10^5^ to 1 × 10^6^ cfu/mL to deliver the required estimated, retained, inhaled, dose of 10–50 cfus to the lungs of each animal. The suspension of Mtb in the nebuliser was plated onto Middlebrook 7H11 OADC selective agar to measure the concentration and confirm retrospectively that the expected doses had been delivered (30).

Within this study, six guinea pigs were euthanised at each of day 5 and 10 days post-infection (dpi) by an overdose of sodium pentobarbital via the intraperitoneal route. Archived, FFPE lung tissues came from previously performed studies whereby the same strain of guinea pigs received the same dose, route of delivery and batch Mtb H37Rv but were euthanised at 3 weeks (*n* = 8), 4 weeks (*n* = 8), and 8 weeks (*n* = 8) post-infection. Guinea pigs necropsied at 24–27 weeks post-infection (wpi) (*n* = 20: *n* = 2,167 dpi; *n* = 1,169 dpi; *n* = 1,178 dpi; *n* = 14,188 dpi; *n* = 2,189 dpi), were given once daily (Monday to Friday) oral treatment of Rifampicin (10 mg/Kg) and Isoniazid (50 mg/Kg) drug regimen between 3–7 wpi to reduce bacterial load and induce relapse of disease between weeks 7 and 28 wpi.

### 2.4. Bacteriology

At necropsy, representative tissue from the lung of each animal (left: cranial lobe; right: cranial lobe and caudal lobe) were removed and placed into sterile Precellys tubes containing ceramic zirconium oxide beads were homogenised in 5 mL of phosphate buffered saline (PBS). Serial dilutions were plated (0.1 mL per plate, in duplicate) onto Middlebrook 7H11 OADC selective agar. After up to 4 weeks of incubation at 37°C, bacterial colonies were counted and duplicate data averaged to measure cfu/mL of viable Mtb in each lung sample homogenate. Where no colonies were observed, a minimum detection limit was set by assigning an average count of 0.5 colonies, equating to 5 cfu/mL.

### 2.5. Histopathology

At necropsy, lungs were removed and fixed by immersion in 10% neutral-buffered formalin [NBF] (Solmedia Ltd., Shrewsbury, United Kingdom). Tissue from pre-defined areas of each lung lobe (left: middle and caudal lung lobes; right: middle and accessory lung lobes) were sampled using a standard protocol to ensure consistency, routinely processed and embedded in paraffin blocks. All tissue blocks were sectioned at 4 μm and stained with haematoxylin and eosin (H&E).

Additionally, serial sections were stained with Ziehl-Neelsen (ZN) technique for the identification of acid-fast bacilli (AFB). Numbers of AFBs were quantified by light microscopy from ZN-stained slides, as previously described by Garcia-Jimenez et al. (2012) (8). The total number of AFBs present in each granuloma was counted and recorded using a scoring system as follows: 0 = no AFBs, 1 = 1–10 AFBs, 2 = 11–50 AFBs, and 3 ≥ 50 AFBs.

Martius Scarlet Blue (MSB) staining was performed on representative tissue sections containing lesions of different developmental stages to evaluate the presence of fibrous tissue. In sections where focally extensive pyogranulomatous lesions were observed, additional Gram staining was carried out to detect non-mycobacterial colonies.

H&E stained slides were scanned with a Hamamatsu S360 (Hamamatsu Photonics, Shizuoka, Japan) digital scanner and e-slides were evaluated using ndp.view2 software (v 2.9.29) (Hamamatsu Phonics, Japan). Slides stained with ZN or MSB special stains were evaluated using light microscopy.

### 2.6. Immunohistochemistry

Immunohistochemical staining (IHC) was performed to study different cell populations within the lung granulomas, including macrophages (Iba-1), myeloid cells (calprotectin positive; MAC387), heterophils (myeloperoxidase positive; MPO), B-cells (Specific Activator Protein; Pax-5) and T cells (CD3). Selected tissue sections from lungs at each time point were selected apart from those at 5 and 10 dpi, due to the very low number of lesions present. At 3, 4 and 8 wpi, tissues from three animals per group were included, whereas from 24–27 wpi, tissues from a total of 11 animals were included. Details of primary antibodies and IHC methods are summarised in Table 1. Briefly, in all cases, deparaffinisation and heat-induced epitope retrieval were performed on the Leica BOND-RXm using BOND Epitope Retrieval Solution 1 (ER1, pH 6.0) for 20 min at 95°C. After primary antibody incubation, immunostaining was performed with the Dako Real EnVision Detection System Peroxidase/DAB, Rabbit/Mouse (Agilent, CA, United States) and counterstained with Gill’s haematoxylin. Finally, slides were routinely dehydrated and mounted using the Ecomount medium (Biocare Medical, CA, United States). Negative controls, consisting of replacement of primary antibody by blocking solution, were included in each run to detect potential non-specific binding. Likewise, IHC stained slides were scanned and subjected to digital image analysis to calculate the percentage of positively stained area in each granuloma (excluding the necrotic cores in all stains except for the MPO IHC runs) by using Nikon NIS-Ar software (Nikon Instruments Inc., NY, United States).

**Table 1:** Summary of immunohistochemical methods: primary antibody details, source, dilution, and blocking solution.

Specificity/Clone	Type of antibody	Source	Blocking solution	Dilution
IBA1	mAb (clone, GT10312)	Invitrogen, MA, United States	Superblock^1^	1:100^2^
MAC387	pAb	Bio-techne, OX, United Kingdom	10% NGS	1:100^2^
MPO	pAb	Invitrogen, MA, United States	Superblock^1^	1:100^2^
CD3	mAb (clone, LN10)	Leica Microsystems, MK, United Kingdom	Superblock^1^	1:50^3^
B-Cell-SAP	mAb (clone, DAK-Pax5)	Agilent, CA, United States	Superblock^1^	1:50^3^

1 Superblock (TBS) Blocking Buffer (Thermo Scientific, United States).

2 Primary antibody incubation 1 h in the oven at 37°C.

3 Primary antibody incubation overnight at 4°C.

MAb, monoclonal antibody; pAb, polyclonal antibody; NGS, normal goat serum; MPO, myeloperoxidase; B-cell SAP, B-cell Specific Activator Protein.

Histopathological and immunohistochemical evaluations were carried out in a ISO9001:2015 and GLP compliant laboratory and evaluated by two veterinary pathologists blinded to the animal details and methodology.

### 2.7. Statistical analysis

Differences between granuloma stages and time point were evaluated for statistical significance, which was set at 0.05, using the *X-sq* for trend test. Figures and data analyses were performed using GraphPad Prism 9.0 software (GraphPad Prism software 9.0, Inc., San Diego, CA, United States).

## 3. Results

### 3.1. Description of granuloma stages (I–IV)

In the lung, granulomatous lesions were differentiated into four distinct stages, broadly reflecting a temporal pattern of lesion development (Table 2). Lesions were located randomly within the parenchyma, or in the fibrous broncho-vascular connective tissue surrounding blood vessels and airways. In general, there was a tendency for granuloma size to increase through progression of the stages. A description of the morphological appearance of each stage is given below:

**Table 2:** Total number of granulomas observed at each stage and time point in guinea pig lungs.

Days/weeks post-inoculation	Granuloma stage				Total count
	I	II	III	IV	
5 dpi (*n* = 6)	12	0	0	0	12
10 dpi (*n* = 6)	9	0	0	0	9
3 wpi (*n* = 8)*	9	28	14	0	51
4 wpi (*n* = 8)*	126	130	37	0	293
8 wpi (*n* = 8)*^,^**	185	209	8	16	418
24–27 wpi (*n* = 20)*^,^**	346	458	48	40	892

dpi, days post infection; wpi, weeks post infection. *X-sq for trend *p* < 0.05 compared to 5 and 10 dpi and **X-sq for trend *p* < 0.05 compared to 3 and 4 wpi.

**Figure 1:**
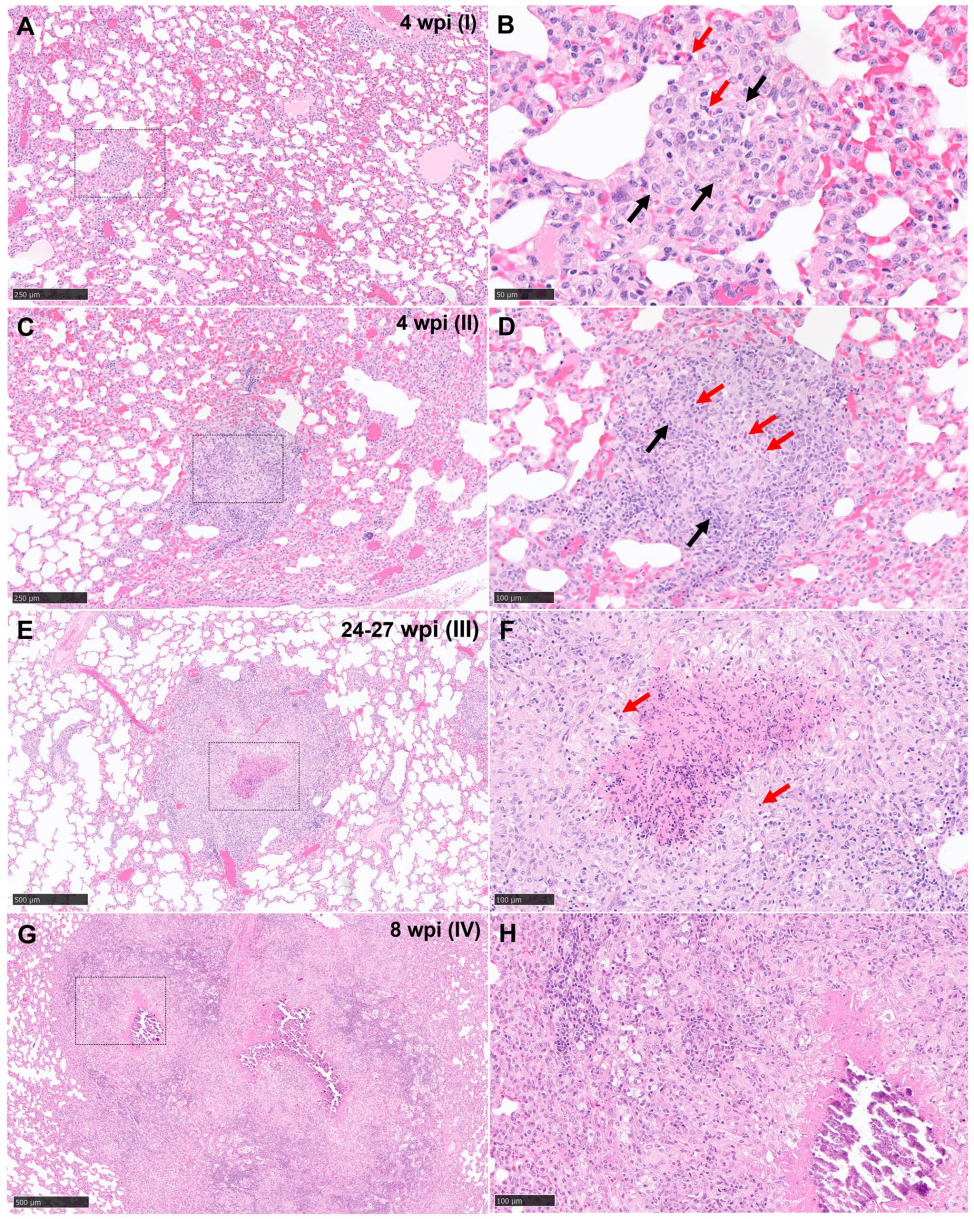
Representative microphotographs of the four stages of tuberculous granulomas in lungs from guinea pigs (H&E). (**A, B**) Stage I. Small with epithelioid macrophages (black arrows) with interspersed lymphocytes and heterophils (red arrows). (**C, D**) Stage II. Organised granulomas with heterophils (red arrows) in the centre and surrounded by epithelioid macrophages interspersed with polymorphonuclear cells (black arrows). (**E, F**) Stage III. Organised, with initial to caseous necrosis with degenerated and/or viable heterophils (red arrows). (**G, H**) Stage IV. Organised, advanced tuberculous lesion with extensive caseous necrosis and mineralisation. Scale bars = 250 μm (insets B, D, F, and H = 100 μm).

**Figure 2:**
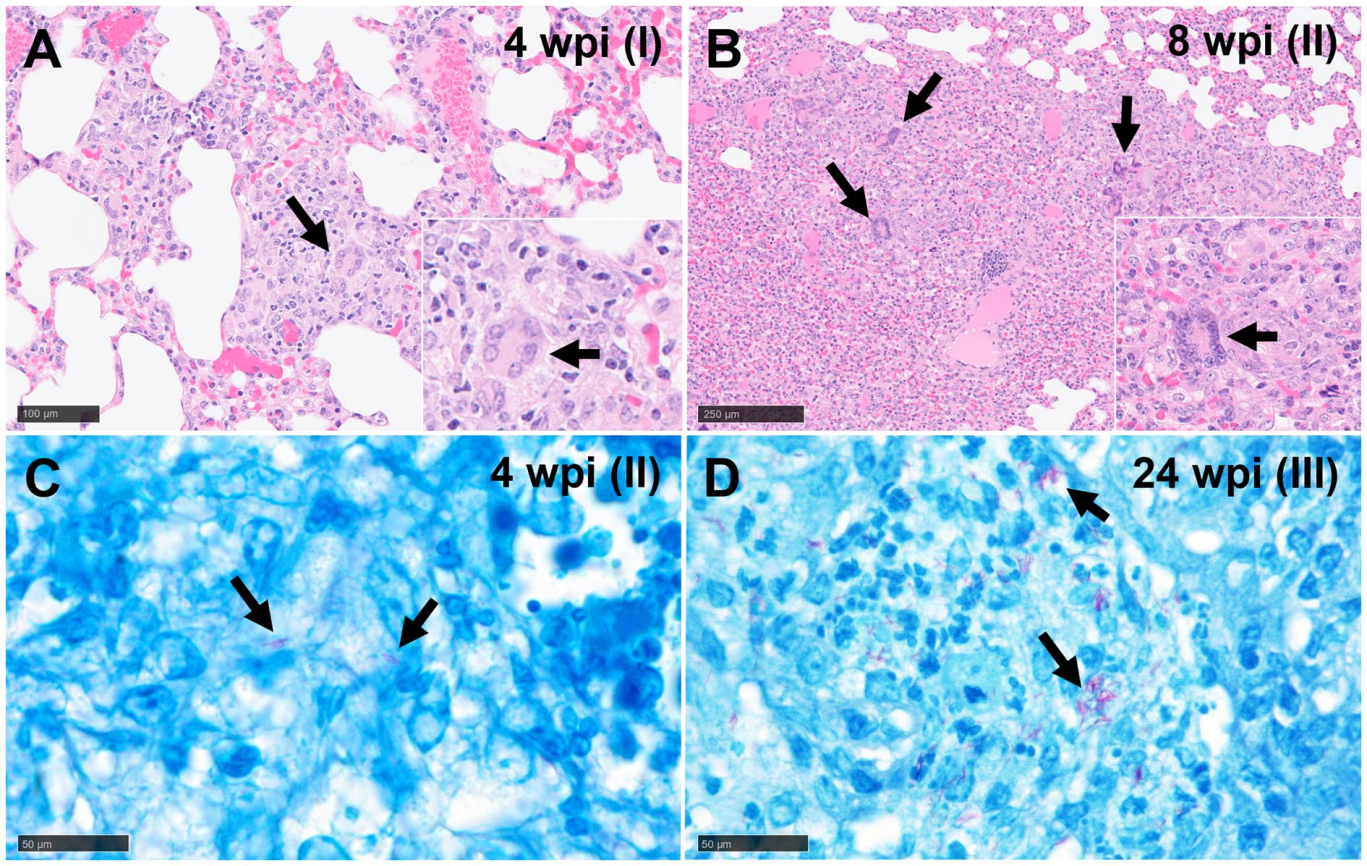
Representative microphotographs of multinucleated giant cells (MNGCs) (H&E) and Ziehl-Neelsen (ZN) staining. **(A)** Epithelioid macrophages forming an initial MNGC (arrows) in a stage I granuloma at 4 wpi and **(B)** several MNGCs (arrows) in a stage II granuloma at 8 wpi. **(C)** Acid-fast bacilli (AFBs, arrows) in lungs of guinea pigs with stage II (4 wpi) and **(D)** stage III (24 wpi) tuberculous granuloma (ZN staining). Scale bars A and B = 100 μm (insets, and C and D = 50 μm).

**Stage I**: these lesions comprised small, unorganised structures which lacked a distinct, circumscribed appearance, with borders that were poorly demarcated from the surrounding parenchyma. They consisted primarily of macrophages and interspersed lymphocytes, with occasional, scattered heterophils and eosinophils; these cells expanded alveolar walls and infiltrated alveolar spaces and/or broncho-vascular connective tissue. In some stage I granulomas, epithelioid macrophages (Figures 1A,B) and few Langhan’s type multinucleated giant cells (MNGCs) were also observed (Figure 2A).

**Stage II**: these lesions were often larger than stage I, circumscribed, and contained well-demarcated borders. They represented a variable morphology, from round to any shape, that included poorly organised or non-necrotic granulomas with initial signs of organisation. Less organised stage II granulomas comprised primarily of scattered lymphocytes and macrophages, the latter often epithelioid, alongside variable numbers of centrally located heterophils (Figures 1C,D). Other more organised granulomas showed a prominent peripheral rim of lymphocytes surrounding a central core of macrophages and variable numbers of heterophils. The majority of heterophils observed were viable, although a small number of degenerate cells were noted in the centre of the lesions. Occasional MNGCs were also present (Figure 2B).

**Stage III**: central necrosis, often mild to moderate, was the key feature observed in stage III lesions. This was characterised by numerous degenerate heterophils with concomitant nuclear pyknosis and karyorrhexis. Variable amounts of early, caseous necrosis were also presented, predominantly comprising homogenous, eosinophilic material. The necrotic core was surrounded by a rim of inflammatory cells, containing variable numbers of heterophils and encircled by epithelioid macrophages and variable numbers of MNGCs. Lymphocytes and plasma cells constituted the outer peripheral rim (Figures 1E,F).

**Stage IV**: these granulomas exhibited extensive, central, caseous necrosis and concomitant, dystrophic calcification (Figures 1G,H). The surrounding tissue comprised primarily of epithelioid macrophages and some lymphocytes. MNGCs were noted variably.

**Figure 3:**
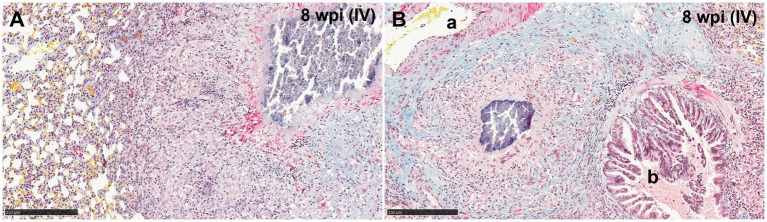
Representative microphotographs of Martius Scarlet Blue (MSB; blue for collagen) staining in lungs of guinea pigs with tuberculous granulomas. **(A)** Stage IV granuloma within the lung parenchyma, not showing a fibrotic capsule surrounding the lesion. **(B)** Stage IV granuloma located between an artery **(A)** and a bronchiole **(B)** showing normal collagen (blue stain) from the lung around the granuloma. Scale bars = 250 μm.

Reactive fibroplasia, with the formation of a fibrous capsule, was not observed in the examined lung tissue (Figure 3A). Occasionally, in some granulomas that developed adjacent to airways or blood vessels, pre-existing broncho-vascular connective tissue was incorporated into the granuloma structure, as confirmed by MSB staining (Figure 3B). This feature was also observed in granulomas located close to the pleural membrane, irrespective of the stage of the granuloma. Furthermore, local expansion of the pleura by inflammatory cells, was also noted. Gram staining did not reveal the presence of non-mycobacterial bacteria in the severe extensive lesions observed in some animals.

### 3.2. Time course development of disease

**Figure 4:**
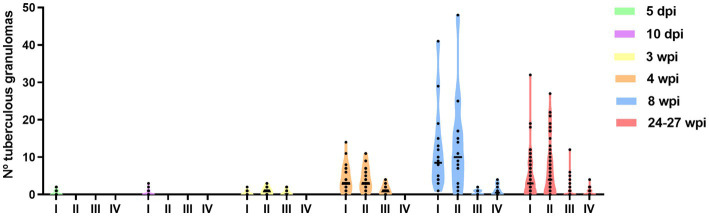
Number of different granulomas from different developmental stages (I–IV) observed in guinea pig lungs at different time points after inoculation (from 5 dpi to 24–27 wpi). Each dot represents a lung section and bars represent median values.

A temporal association was noted between the progression in the stage of lesion development and an increase in the total number of granulomas observed in the lung (Figure 4). The total number of granulomas at each developmental stage for each time point is summarised in Table 2 and plotted in Figure 4. At 5 and 10 dpi, only stage I granulomas were present (*n* = 12 and *n* = 9 respectively), representing early-stage granuloma formation. By contrast, at 3 wpi, the majority of granulomas were stage II (*n* = 28), with fewer numbers of stage III (*n* = 14) and stage I granulomas (*n* = 9). By 4 wpi, there were abundant stage I granulomas (*n* = 126) together with numerous stage II (*n* = 130) and lesser numbers of stage III granulomas (*n* = 37). A predominance of stage I (*n* = 185) and stage II (*n* = 209) granulomas were noted by 8 wpi. In addition, there were a small number of stage IV granulomas with partially to fully mineralised necrotic centres (*n* = 16), as well as stage III granulomas (*n* = 8). At the final time point of 24–27 wpi, some lung lobes showed extensive, coalescing pneumonia and marked consolidation affecting the majority of the parenchyma. Most of the granulomas observed were stage II (*n* = 458); abundant stage I granulomas were also noted (*n* = 346) and a smaller number of stage III (*n* = 48) and stage IV granulomas (*n* = 40).

The presence and frequency of AFB in each granuloma stage at different time points are summarised in Table 3. In the early time points (5 and 10 dpi), AFBs were not detected in the small number of observed lesions. However, by 3 and 4 wpi, AFBs were noted in stage II (Figure 2A) and III granulomas with a tendency for higher numbers of AFBs visible in stage III (Figure 2B); stage I lesions did not show any AFB at these time points. By 8 wpi, AFB were observed in low numbers in a small proportion of stage I and II granulomas. However, the majority of stage III and IV granulomas showed abundant AFBs. At 24–27 wpi, abundant AFBs were observed in all granuloma stages.

**Table 3:** Total score for the number of AFB in granulomas at each stage and time point in the guinea pig lungs.

Granuloma stage	5 dpi				10 dpi				3 wpi				4 wpi				8 wpi				24–27 wpi			
	0	1	2	3	0	1	2	3	0	1	2	3	0	1	2	3	0	1	2	3	0	1	2	3
Stage I	6	0	0	0	3	0	0	0	2	0	0	0	5	0	0	0	21	2	0	0	27	11	5	0
Stage II	0	0	0	0	0	0	0	0	0	2	1	0	2	8	0	0	17	9	0	0	24	23	3	10
Stage III	0	0	0	0	0	0	0	0	0	0	0	2	0	2	4	7	0	1	1	0	2	10	3	1
Stage IV	0	0	0	0	0	0	0	0	0	0	0	0	0	0	0	0	0	3	3	3	1	2	3	0
Total count	6	0	0	0	3	0	0	0	2	2	1	2	7	10	4	7	38	15	4	3	54	46	14	11

The total number of AFBs present in each granuloma were counted and recorded using a scoring system: 0 = no AFBs, 1 = 1–10 AFBs, 2 = 11–50 AFBs, and 3 ≥ 50 AFBs. AFB, acid fast bacilli; dpi, days post infection; wpi, weeks post infection.

### 3.3. Viable *Mycobacterium tuberculosis* in lung

**Figure 5:**
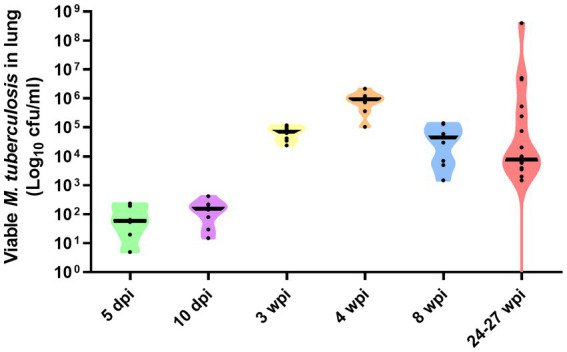
Number of viable Mtb H37Rv in lung at day 5 and 10, and weeks, 3, 4, and 8 weeks post-infection by the aerosol route of delivery. Bacterial load in lungs at 24–27 weeks post-infection represent the result of relapse of disease following sub-optimal delivery of drug treatment delivered between weeks 3–7 post-infection. Individual dots represent each animal in each group. Viable bacterial load are expressed as Log_10_ cfu/mL, each animal in the group represented as black circles, and group mean expressed as horizontal black bars, in violin plots.

Bacterial load data measured as cfu/mL of *M. tuberculosis* measured in lung at 5 and 10 days, and 3, 4, 8, and 24–27 weeks post-infection, is represented in Figure 5. Although at 5 and 10 dpi expected low values (around 10^2^ cfu/mL) of bacterial burden were detected, at 3 wpi, numbers of viable bacilli had more than doubled (around 10^5^ cfu/mL). Peak bacterial loads were detected at 4 wpi with values around 10^6^ cfu/mL. Similar values than found at 3 wpi were found at 8 wpi to increase progressively until the end of the study (10^7^ cfu/mL). A greater range of bacterial load (averaging at around 10^4^ cfu/mL) were observed in animals at 24–27 weeks, following relapse from sub-optimal drug treatment (Figure 5).

### 3.4. Distribution of cell populations within granulomas

**Figure 6:**
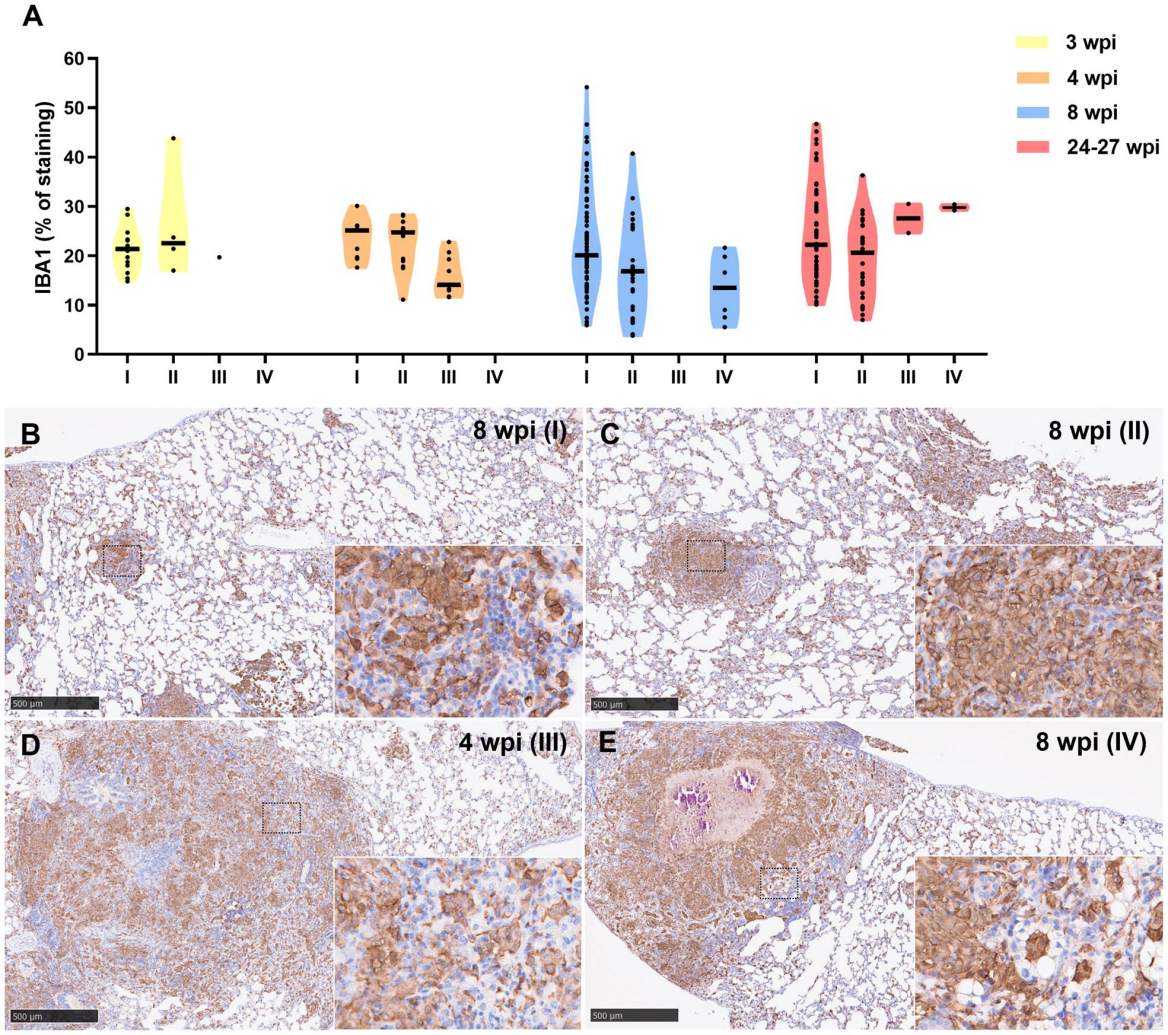
Iba1+ immunohistochemical detection in stage I, II, III, and IV granulomas throughout the experiment. **(A)** Percentage of Iba1^+^ staining in stage I to IV granulomas at 3, 4, 8, and 24–27 wpi. Dots show the individual percentage of staining in each granuloma. Lines show the median value of the percentage of staining in the different stages throughout the study. **(B)** Iba1 expression in stage I granuloma at 8 wpi. **(C)** Iba1 expression in stage II granuloma at 8 wpi. **(D)** Iba1 expression in stage III granuloma at 4 wpi. **(E)** Iba1 expression in stage IV granuloma at 8 wpi. Insets show close-up images at higher magnification showing Iba1^+^ macrophages. Scale bars = 500 μm (insets = 100 μm).

Iba1 was the most predominant marker in all stages of granulomas throughout the experiment (Figure 6). The staining was detected on the cell membrane of macrophages, including epithelioid and foamy macrophages and MNGCs, and diffusely extended in all the lesions, mainly from stage I and stage II granulomas (Figures 6B,C). In stage III and IV granulomas, the staining was also demonstrated in epithelioid macrophages surrounding the necrotic cores and central calcification, as well as in foamy macrophages at the periphery of the lesions (Figures 6D,E). No significant differences were observed in the percentage of Iba1+ staining between the different stages throughout the study.

**Figure 7:**
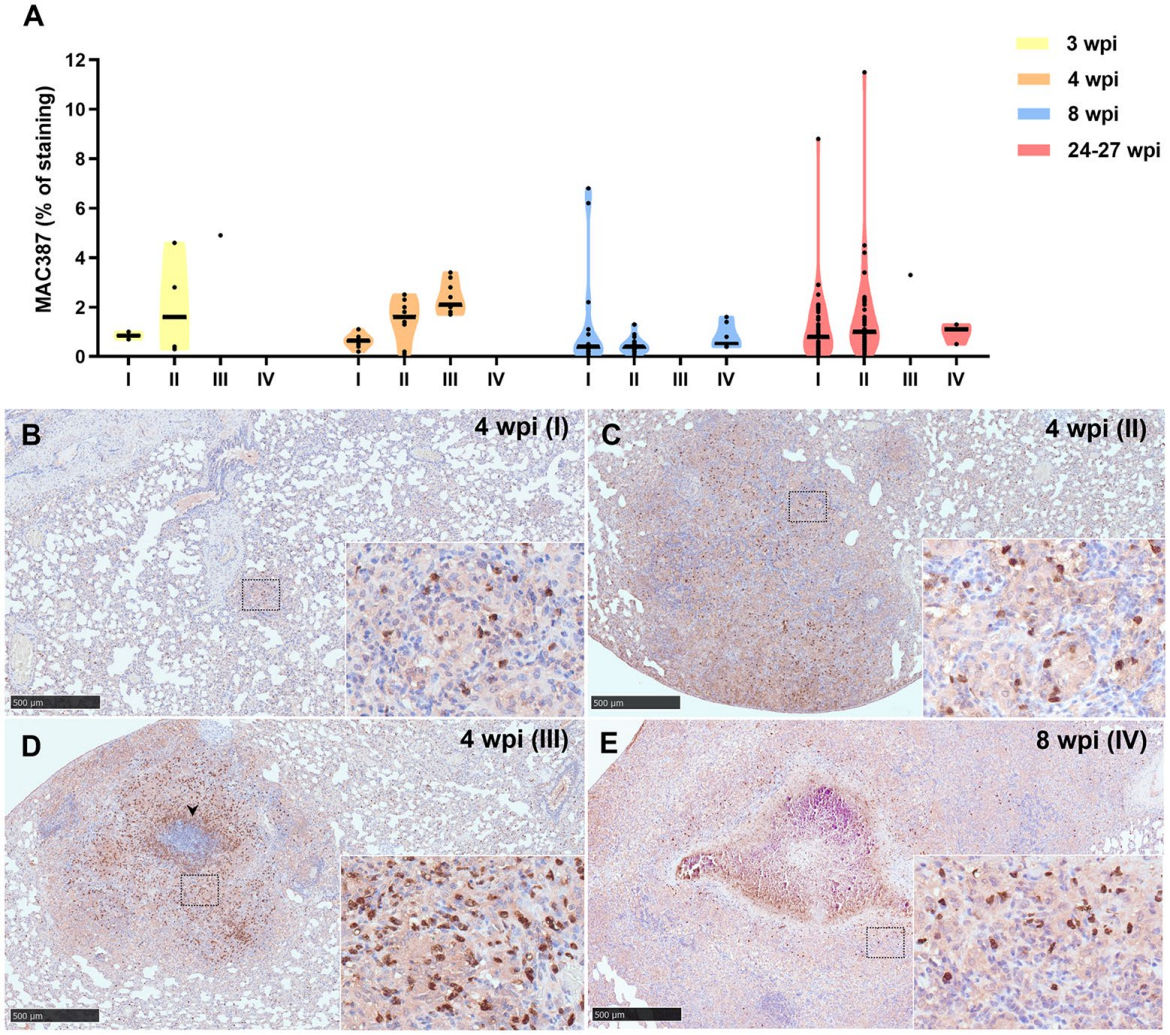
MAC387+ immunohistochemical detection in stage I, II, III, and IV granulomas throughout the experiment. **(A)** Percentage of MAC387^+^ cells in stage I to IV granulomas at 3, 4, 8, and 24–27 wpi. Dots show the individual percentage of staining in each granuloma. Lines show the median value of the percentage of staining in the different stages throughout the study. **(B)** MAC387 expression in stage I granuloma at 4 wpi. **(C)** MAC387 expression in stage II granuloma at 4 wpi. **(D)** MAC387 expression surrounding a necrotic core (arrowhead) in stage III granuloma at 4 wpi. **(E)** MAC387 expression in stage IV granuloma at 8 wpi. Insets show close-up images at higher magnification showing MAC387^+^ cells: macrophages and polymorphonuclear cells. Scale bars = 500 μm (insets = 100 μm).

Results from MAC387 positive staining quantification are represented in Figure 7A. MAC387+ staining was detected in the cytoplasm of scattered macrophages and granulocytes within the tuberculous granulomas (Figures 7B–E). An increase in MAC387+ staining was observed coinciding with the progression of the lesion, being the maximum percentage observed in stage III (Figures 7A,D). In this stage, MAC387+ cells formed a rim of inflammatory cells surrounding the necrotic core (Figure 7D). These differences were specially observed at 3 and 4 wpi (Figure 7A). At 8 and 24–27 wpi, no significant differences were found in the percentage of staining between the different stages of granulomas.

**Figure 8:**
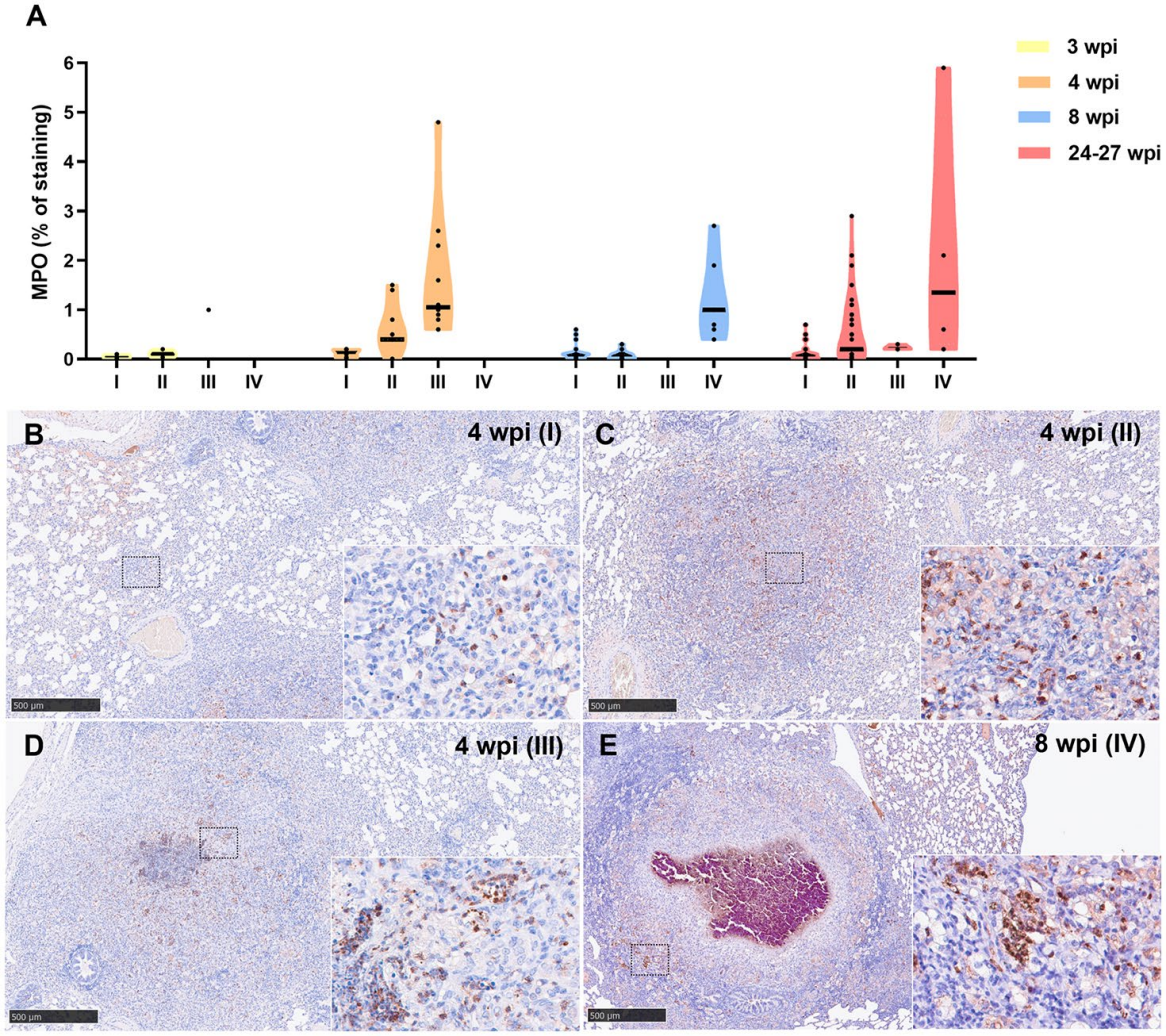
Myeloperoxidase (MPO) + immunohistochemical detection in stage I, II, III, and IV granulomas throughout the experiment. **(A)** Percentage of MPO^+^ staining in stage I to IV granulomas at 3, 4, 8, and 24–27 wpi. Dots show the individual percentage of staining in each granuloma. Lines show the median value of the percentage of staining in the different stages throughout the study. **(B)** MPO expression in stage I granuloma at 4 wpi. **(C)** MPO expression in stage II granuloma at 4 wpi. **(D)** MPO expression in stage III granuloma at 4 wpi. **(E)** MPO expression in stage IV granuloma at 8 wpi. Insets show close-up images at higher magnification showing MPO^+^ polymorphonuclear cells. Scale bars = 500 μm (insets = 100 μm).

Similar kinetic expression pattern was observed for the myeloperoxidase (MPO) marker (Figure 8A). However, MPO was also highly expressed in stage IV granulomas at the late time points (8 and 24–27 wpi) (Figure 8E). MPO was expressed in scattered granulocyte-like cells of stage I and II granulomas (Figures 8B,C), as well as in the necrotic areas and inflammatory infiltrates of stage III and IV granulomas (Figures 8D,E).

**Figure 9:**
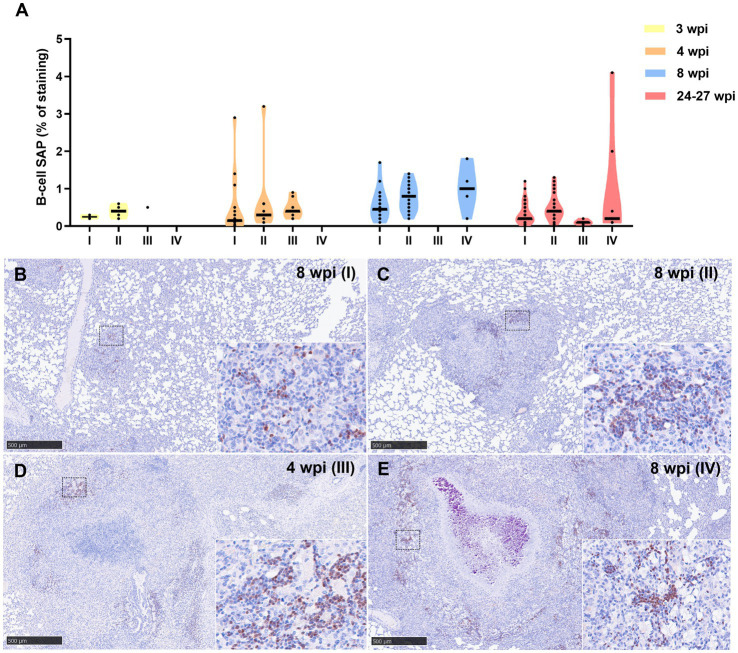
B-cell Specific Activator Protein+ (SAP+) immunohistochemical detection in stage I, II, III, and IV granulomas throughout the experiment. **(A)** Percentage of B-cell SAP+ staining in all stages granulomas at 3, 4, 8, and 24–27 wpi. Dots show the individual percentage of staining in each granuloma. Lines show the median value of the percentage of staining in the different stages throughout the study. **(B)** B-cell SAP expression in stage I granuloma at 8 wpi. **(C)** B-cell SAP expression in stage II granuloma at 8 wpi. **(D)** B-cell SAP expression in stage III granuloma at 4 wpi. **(E)** B-cell SAP expression in stage IV granuloma at 8 wpi. Insets show close-up images at higher magnification showing B cell SAP^+^ lymphocytes. Scale bars = 500 μm (insets = 100 μm).

Results from B-cell marker quantification are represented in Figure 9A. At 3, 4, and 8 wpi, a progressive increase in the percentage of this immunostaining was observed from stage I to stage IV (Figure 9). Higher expression of this marker was detected at 8 wpi in comparison with 4 and 24–27 wpi in stage I and II granulomas. Positive staining to B-cell SAP was found in the nuclei of B lymphocytes within multifocal inflammatory infiltrates in all granuloma stages (Figures 9B–E). In stage III and IV granulomas, the B-cell SAP^+^ infiltrates were present in the outer rims of the granulomas and within the stage I and II “satellite” granulomas surrounding the larger lesions (Figures 9D,E).

**Figure 10:**
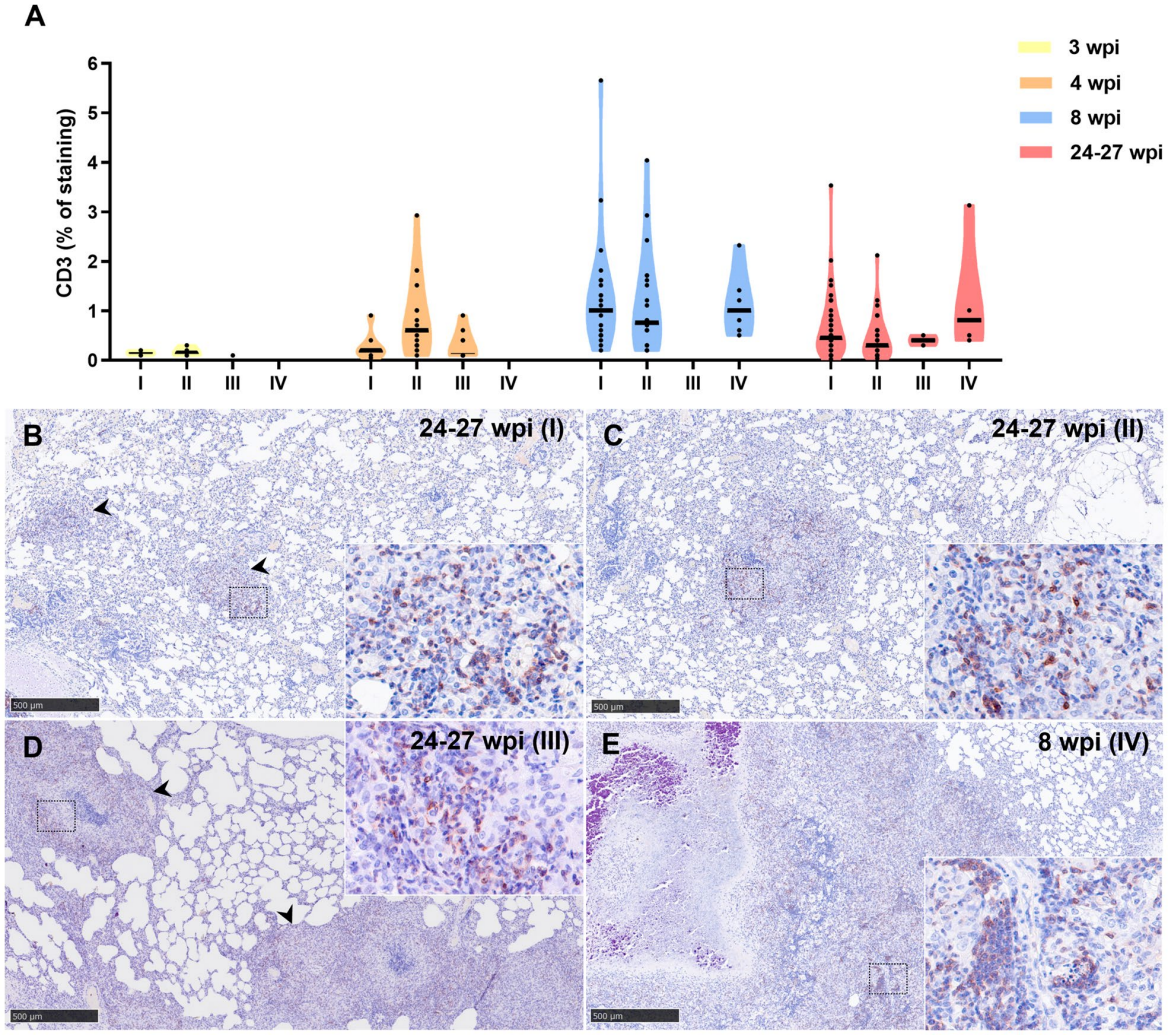
CD3+ immunohistochemical detection in stage I, II, III, and IV granulomas throughout the experiment. **(A)** Percentage of CD3^+^ staining in stage I to IV granulomas at 3 to 24–27 wpi. Dots show the individual percentage of staining in each granuloma. Lines show the median value of the percentage of staining in the different stages throughout the study. **(B)** CD3 expression in stage I granuloma (arrowheads) at 24–27 wpi. **(C)** CD3 expression in stage II granuloma at 24–27 wpi. **(D)** CD3 expression in stage III granuloma (arrowheads) at 24–27 wpi. **(E)** CD3 expression in stage IV granuloma at 8 wpi. Insets show close-up images at higher magnification showing CD3+ T lymphocytes. Scale bars = 500 μm (insets = 100 μm).

Lower percentage of CD3+ staining was detected at 3 wpi in all granuloma stages compared to 4, 8, and 24–27 wpi, being the maximum expression observed at 8 wpi (Figure 10A). At 8 and 24–27 wpi, similar expression of CD3+ staining was detected in the different granuloma stages (Figure 10A). The staining was detected in the cytoplasm and cell membrane of T lymphocytes diffusely scattered within stage I and II granulomas and located at the periphery of stage III and IV granulomas (Figures 10B–E).

## 4. Discussion

The results describe the morphological features observed in the formation and progression of tuberculous granulomas in the lungs of guinea pigs and provide a clearly defined methodology for their classification into stages. These stages can be used to assess the severity and progression of tuberculosis in this animal model and aid in the evaluation of therapeutics and prophylactics against human disease.

The guinea pig model is widely used in tuberculosis research due to its similarities with human disease, specifically regarding disease progression and immunopathology (11). The pathology of tuberculous granuloma formation in the lung of the guinea pig has already been well documented in the literature (11, 12, 20, 30, 32–35). Microscopic evaluation of morphological changes using subjective scoring methodologies have been used for many years to evaluate disease severity and progression in multiple, pre-defined lung lobes; these include assessment of the percentage of lung parenchyma affected by tuberculous granulomatous inflammation, ranging from multifocal, discrete granulomas to consolidative changes, as well as microscopic features such as presence/absence of necrosis, dystrophic calcification and extent of lymphocytic infiltration of granulomas (36). Outputs, such as mean scores from lung lobes of individual animals, as well as group mean scores, can be compared alongside bacterial, immunological and other relevant data to discriminate between test and control groups. Similar scoring systems have been devised in other animal models of tuberculosis, such as macaques (15, 22, 37, 38), cattle (13, 39–41), badgers (42, 43), deer (8), swine (44), or mice (45). The current study aimed to enhance histopathological outputs through the provision of a semi-quantitative method characterising granuloma morphology. This approach provides additional discriminatory power, helping to overcome potential limitations associated with semi-quantitative scoring, such as experience and personal bias of the pathologist.

The granuloma developmental stages described here are similar to those described in cattle, from stage I (initial) to stage IV (necrotic and mineralised) (13, 39–41). The cellular composition of the granulomas is also similar to what is observed in other animal models of MTBC infection, with heavy presence of activated macrophages from the early stages, polymorphonuclear cells (neutrophils or heterophils), and rims of T and B cells surrounding the necrotic cores in the late stages of granuloma development. A large quantity of heterophils and activated macrophages is observed in late-stage granulomas, with high expression of MPO at the necrotic core and inner layers of the granulomas. The number of AFBs also increase concomitantly with lesion development, as seen in other animal models (37, 40). As expected, the viable bacterial burden in lung measured by plating homogenates onto selective medium, also increased concurrently with increased presence of AFBs and lesion development. The reduction of viable bacteria in lungs between 4 and 8 weeks post-infection is commonly observed, and thought to be related to the switching of innate to adaptive host immune responses (20, 46, 47).

The presence of MNGCs is not prominent and only occasional in some granulomas, in contrast to what is observed in other models such as non-human primates (37, 48) or cattle (40, 41). MNGCs are normally formed by the fusion of macrophages responding to the intracellular persistence of mycobacteria. Their role in the different animal models of TB is under constant review, with some authors showing a possible correlation of vaccine protection with fewer presence of MNGCs (40).

The findings indicated that fibroplasia was not a feature in the formation of pulmonary granulomas in guinea pigs at this exposure dose and time points post-challenge. In general, the prominence of peripheral fibrosis in granuloma development varies between species, with non-human primates or cattle exhibiting prominent fibrous deposition (13, 37, 39–41, 48), whereas being sparse or absent in other animal models like mice, except for specific genetically modified strains (49). This fact represents a limitation of the guinea pig model for testing some potential therapies where fibrosis plays an important role. Conversely, the lack of a fibrotic capsule in late stages granulomas can be an advantage in this animal model for target therapies directed to advanced lesions where a xenobiotic can access the inner layers of the granuloma in the absence of a significant, fibrotic barrier.

Interestingly, the lesion development and cellular composition of granulomas observed in this study show many similarities to pulmonary TB granulomas in human patients, including the presence of solid non-necrotising early lesions to advanced necrotic granulomas categorised in four stages like in cattle and guinea pigs (50).

## 5. Conclusion

The guinea pig model provides a reliable, cost-effective method of evaluating potential new therapeutics and prophylactics in the treatment and prevention of human tuberculosis. This study demonstrates a refinement in the evaluation of the severity and extent of pulmonary tuberculous disease in the guinea pig model through the provision of a quantitative methodology for staging granuloma progression; this, in turn, can provide additional data to aid in the evaluation of future, potential drug and vaccine targets against human tuberculosis. Moreover, the IHC techniques developed may be valuable tools to characterise the local immune responses and lesions in other animal models of disease using guinea pigs.

## Data availability statement

The raw data supporting the conclusions of this article will be made available by the authors, without undue reservation.

## Ethics statement

The animal study was reviewed and approved by UK Health Security Agency, Animal Welfare and Ethical Review body, Porton Down, UK and authorised under an appropriate UK Home Office project license. The study was conducted in accordance with the local legislation and institutional requirements.

## Author contributions

FL-M: Formal analysis, Investigation, Writing – original draft, Writing – review & editing. IR-T: Data curation, Formal analysis, Investigation, Methodology, Validation, Writing – review & editing. LH: Data curation, Formal analysis, Methodology, Writing – review & editing. AB: Data curation, Methodology, Writing – review & editing. IA-R: Data curation, Formal analysis, Writing – review & editing. RW: Formal analysis, Methodology, Writing – review & editing. SC: Conceptualization, Data curation, Formal analysis, Funding acquisition, Project administration, Writing – review & editing. ER: Formal analysis, Validation, Writing – review & editing. FS: Conceptualization, Formal analysis, Funding acquisition, Investigation, Resources, Supervision, Writing – original draft, Writing – review & editing.
